# Massive Scrotal Lymphedema in an Adolescent with Intellectual Disability: A Rare Case of Infection-Related Scrotal Enlargement Requiring Subtotal Scrotectomy

**DOI:** 10.7759/cureus.81821

**Published:** 2025-04-07

**Authors:** Muhammad Saqlain, Masood Ur-Rauf Khan Hiraj, Muhammad Ans, Muhammad Usama Ilyas Ahmed

**Affiliations:** 1 Department of General Surgery, Nishtar Hospital, Multan, PAK

**Keywords:** chronic inflammation, hypertrophy, infection-related lymphedema, reconstruction, scrotal lymphedema

## Abstract

An uncommon disorder marked by aberrant fluid accumulation brought on by lymphatic blockage, scrotal lymphedema presents major functional, cosmetic, and hygiene problems. It can be acquired or congenital; the most often occurring causes are infection, persistent inflammation, and filariasis. This case report describes a 17-year-old male patient with intellectual disability who had severe scrotal lymphedema that gradually developed over four years, causing problems with ambulation and hygienic care. Under a clinical examination, the scrotum (40 x 15 cm) was swollen and expanded, hiding the penis but having palpable testes. Ultrasound verified without testicular involvement scrotal wall thickening. After a subtotal scrotectomy preserves the testes, penis, and spermatic cord, scrotal reconstruction was done considering the functional limitation. Chronic inflammatory alterations without filarial organisms revealed by histopathology suggested an etiology connected to infections. The patient healed postoperatively with better mobility and hygienic standards. This example emphasizes the need for early identification and surgical intervention in controlling large scrotal lymphedema to maximize functional and cosmetic results.

## Introduction

In inadequate lymphatic outflow, lymphedema results from an aberrant accumulation of extracellular fluid in the subcutaneous compartment [1]. An unusual condition called scrotal lymphoedema results from a congested lymphatic system emptying into the scrotum. Though radiation, tumors, or granulomatous illnesses can all aggravate filariasis, the most common cause of scrotal lymphedema is this parasite [2]. Often called "scrotal elephantiasis in severe cases," scrotal lymphedema is a chronic and incapacitating disorder marked by increasing scrotal swelling, thickened skin, and notable functional disability [3]. The etiology can be congenital or acquired; acquired cases arise from infections including lymphogranuloma venereum, tuberculosis (TB), and chronic inflammatory diseases [4]. Additional contributory causes include trauma, radiation treatment, recurrent cellulitis, and blockage resulting from malignancies influencing the pelvic or inguinal lymphatic drainage. Chronic inflammatory processes like recurring bacterial or fungal infections have been linked to increasing lymphatic dysfunction, therefore aggravating the degree of the disease [5]. Scrotal lymphedema without appropriate treatment can cause ulceration, recurring infections, and irreversible tissue alterations, so profoundly compromising the patient's mobility, cleanliness, and psychological well-being. Evaluating the degree of lymphatic block and directing treatment decisions depends much on diagnostic imaging. Commonly employed to evaluate the scrotal and inguinal lymphatics, ultrasonic and Doppler imaging provide important information regarding tissue composition, fluid buildup, and vascular involvement [6]. Confirming the underlying pathology in situations with suspected cancer or parasite infection depends on histological study. Although conservative treatment, including antibiotic prevention, hygienic care, and compression therapy, can help reduce moderate symptoms, advanced cases often call for surgical intervention. In extreme situations, the treatment of choice is a subtotal scrotectomy with reconstruction, meant to restore normal function, improve cosmesis, and raise the general quality of life of the patient [7].

## Case presentation

A 17-year-old male patient with intellectuall disablity presented with a progressively enlarging scrotum over the past five years. The swelling caused significant discomfort, difficulty in ambulation, and impaired hygiene, leading to recurrent episodes of skin infections and ulcerations. The patient did not report any history of trauma, filarial exposure, or prior surgical interventions.

**Figure 1:**
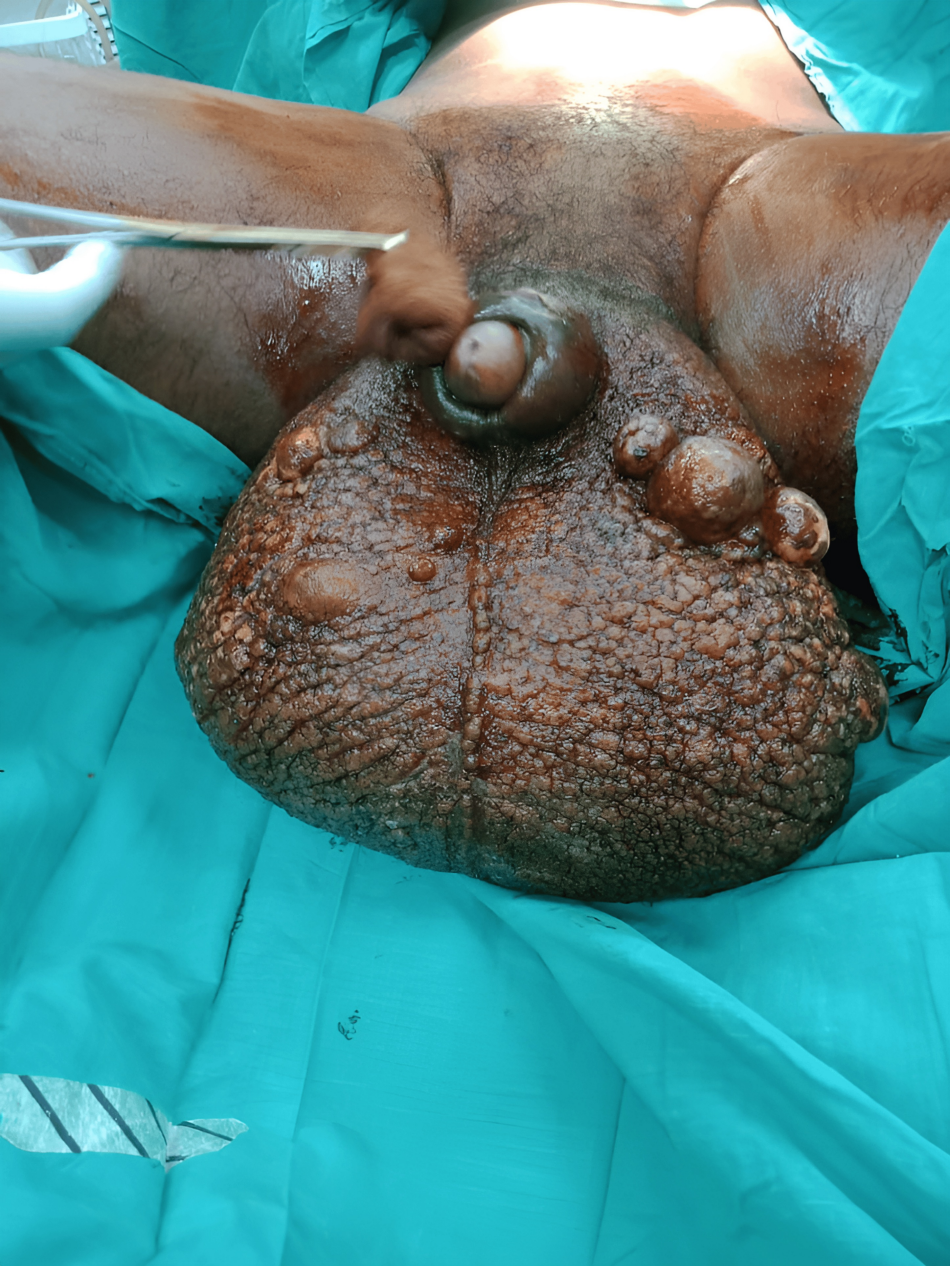
Preoperative image of massive scrotal lymphedema in an adolescent with intellectual disability, showing extensive scrotal enlargement and distorted anatomy due to chronic infection-related lymphedema.

On examination, the scrotum was grossly enlarged, with markedly thickened, hyperpigmented, and indurated skin (Figure 1). The penis was almost completely buried within the edematous scrotal tissue. Multiple areas of lichenification, chronic dermatitis, and ulcerations were noted, particularly in dependent regions. No palpable lymphadenopathy was observed.

**Figure 2:**
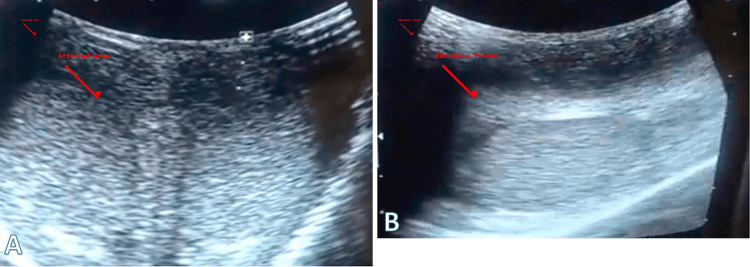
Ultrasound images of the affected scrotal region (A) Diffuse hyperechoic areas indicating chronic inflammatory changes and fibrosis; (B) Fluid collection and edema suggestive of longstanding lymphedema. These findings correlate with the clinical and histopathological features of infection-related lymphedema.

Scrotal ultrasound (Figure 2) showed diffuse thickening and increased echogenicity of the scrotal wall, consistent with chronic inflammatory fibrosis. There was significant subcutaneous fluid accumulation, suggestive of longstanding lymphedema. The testes and epididymis were normal in size, echotexture, and vascularity, with no evidence of intra-testicular masses or hydrocele. These findings supported a diagnosis of chronic infection-related scrotal lymphedema.

**Figure 3:**
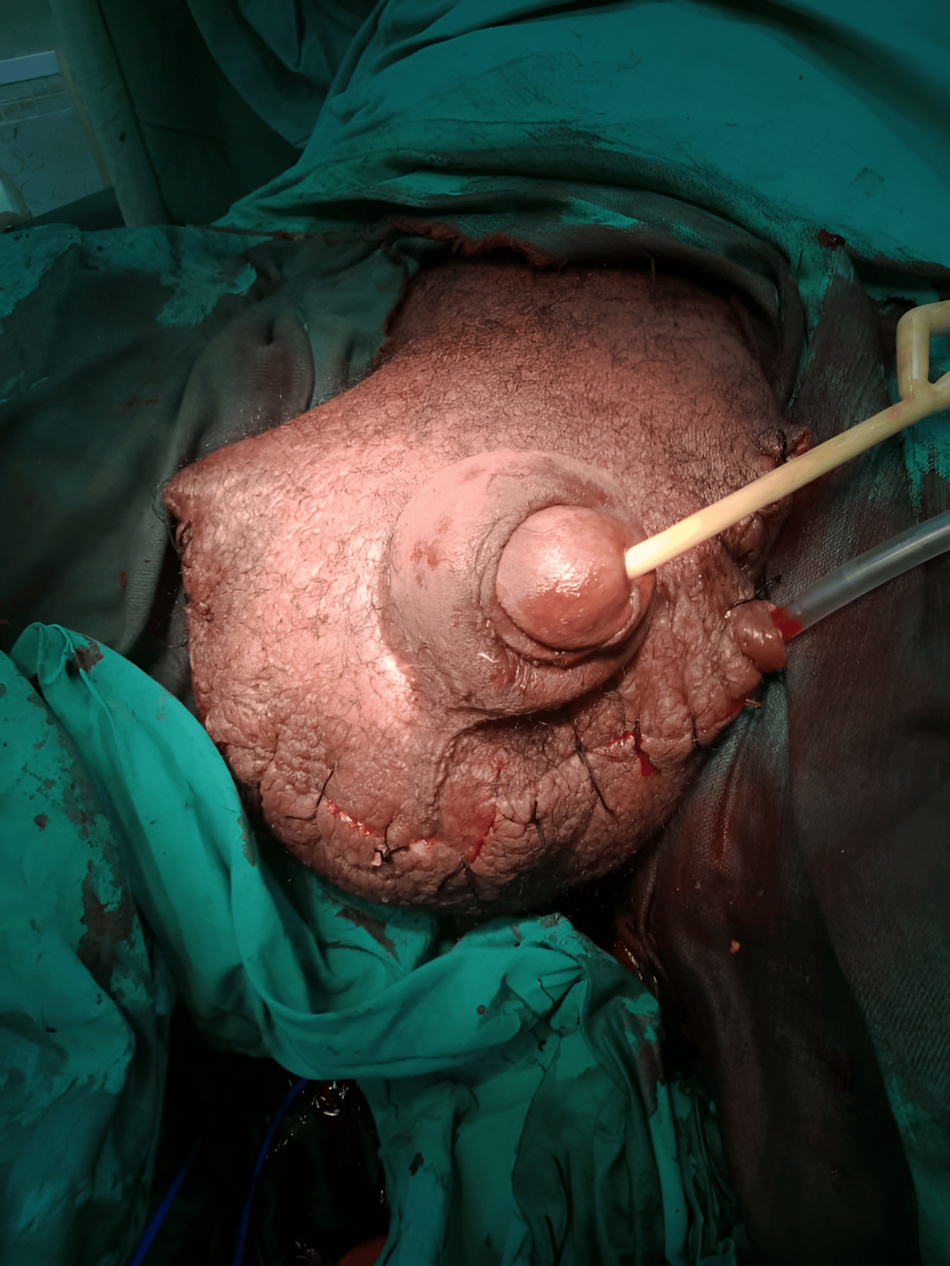
Postoperative image following subtotal scrotectomy, demonstrating partial reconstruction with preserved penile structures and a drain in place for fluid management.

Considering the severity of the condition and its functional impact, the patient underwent subtotal scrotectomy with penile preservation (Figure 3). Intraoperatively, thickened and fibrotic scrotal tissue was excised, preserving the penile structures and ensuring adequate coverage for reconstruction. A closed suction drain was placed to prevent postoperative fluid accumulation.

**Figure 4:**
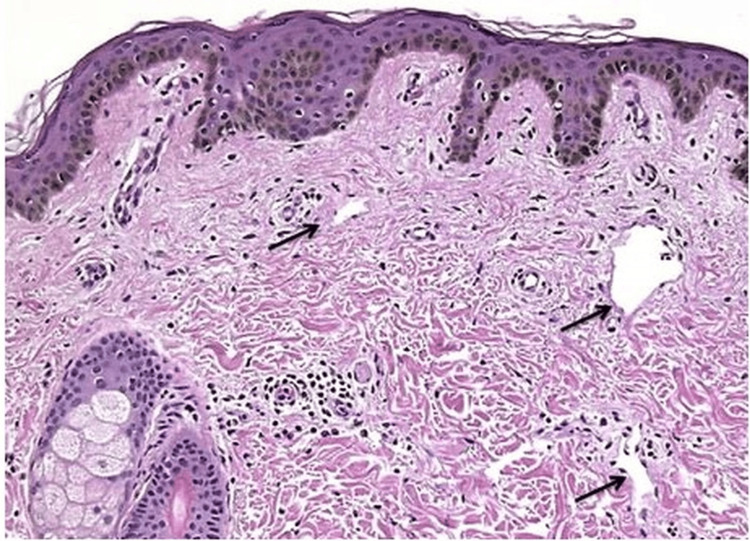
Histopathology image showing chronic inflammatory changes with fibrosis and lymphatic dilation, consistent with infection-related lymphedema.

**Figure 5:**
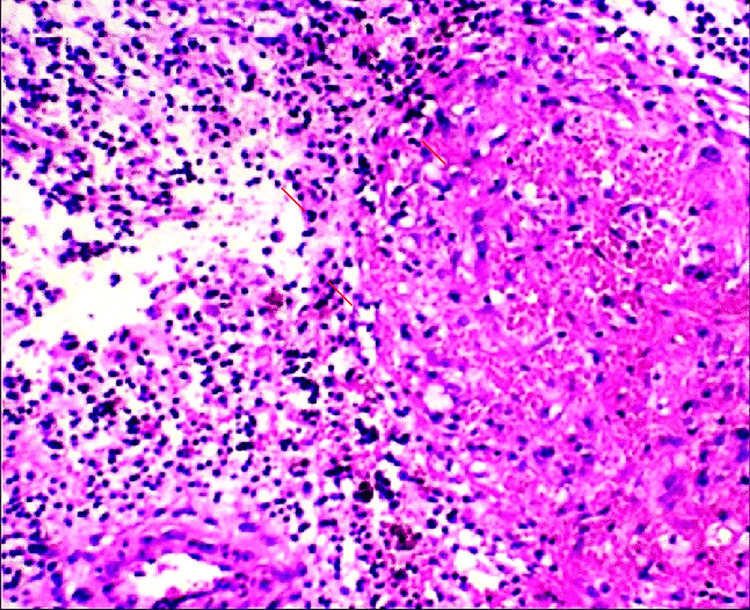
Higher magnification histopathology image demonstrating dense inflammatory infiltrate with no evidence of filarial organisms, confirming the non-parasitic nature of the condition.

Histopathological examination of the resected tissue (Figures 4, 5) revealed dense inflammatory infiltrates, fibrosis, and lymphatic dilation, consistent with infection-induced lymphedema. No evidence of filarial organisms or malignancy was noted, confirming a non-parasitic etiology.

The patient had an uneventful postoperative recovery, with significant relief from discomfort and improved mobility. He was discharged with proper wound care instructions and scheduled for regular follow-ups to monitor healing and prevent recurrence. Table 1 outlines the differential diagnosis considered during the evaluation.

**Table 1:** Laboratory findings prior to surgery

Test	Result	Reference range
White blood cell (WBC) count	12.5 × 10⁹/L	4.0 – 11.0 × 10⁹/L
Erythrocyte sedimentation rate (ESR)	40 mm/hr	0 – 20 mm/hr
​​​​​​​C-reactive protein ( CRP)	15 mg/L	< 10 mg/L
Filarial serology	Negative	Negative
Blood glucose	90 mg/dL	70 – 110 mg/dL

## Discussion

Lymphedema is an abnormal accumulation of protein-rich fluid in soft tissues due to an impaired lymphatic drainage system. The imbalance between lymph production and absorption leads to fluid retention, inflammation, pain, and discomfort [8]. In chronic cases, prolonged lymphatic dysfunction results in fat deposition and fibrosis. The restriction of lymphatic flow further contributes to ductal dilatation, connective tissue overgrowth, interstitial edema, and persistent inflammation [9].

Globally, an estimated 200 million people are affected by lymphedema [10]. The condition may be either congenital or acquired, with the latter being more common. Acquired lymphedema is often secondary to infections, particularly lymphogranuloma venereum and filarial infestations. A major cause of acquired lymphedema in tropical areas, lymphatic filariasis is spread by mosquito bites and linked to parasite infections [11].

Evaluating lymphatic blockage depends much on diagnostic imaging. While vascular Doppler may show lymphatic dilatation, ultrasonic and CT scans might spot extrinsic compression of lymphatic channels. Doppler ultrasounds can also find living parasites in filarial infections. Additional serological testing helps to pinpoint infectious causes, including bloodstream chlamydia and microfilariae. Managing lymphedema is still difficult since treatment mostly aims at symptom control rather than cure. Conventional techniques serve to lower fluid collection and lower the risk of infections by means of compression therapy, manual lymphatic drainage, aerobic activity, and rigorous skin care. Severe situations where functional impairment or cosmetic defect call for tissue removal, reserve surgical intervention. Wound healing slows down postoperatively and requires careful follow-up and extended hospital stays. To maximize results, ideal covering of the excised area may require autologous skin grafts or local flaps [12].

Although treatment can produce good results, ongoing lymphatic blockage makes recurrence a reality, even if it is rare. Reducing problems mostly depends on daily limb hygiene, physical activity, and suitable footwear, among other preventive actions [13]. Controlling lymphatic filariasis has found success using mass drug administration (MDA) using antifilarial drugs such as diethylcarbamazine and ivermectin. Still vital in stopping disease development are public health campaigns emphasizing early diagnosis, patient education, and lifestyle changes. Notwithstanding continuous difficulties, developments in multidisciplinary treatment strategies and research help to improve general illness management and patient outcomes.

## Conclusions

Scrotal lymphedema, though rare, poses significant challenges in terms of physical, psychological, and functional well-being, particularly in patients with limited self-care capacity. Early identification and prompt intervention are crucial in preventing complications such as infections and mobility restrictions. This case highlights the importance of a multidisciplinary approach, incorporating diagnostic imaging, histopathology, and appropriate surgical management. Subtotal scrotectomy with scrotal reconstruction proved effective in improving both mobility and hygiene for the patient. Infections, chronic inflammation, and lymphatic dysfunction remain key factors in the etiology of scrotal lymphedema, underscoring the need for early intervention. Long-term management should focus on patient education, infection prevention, and maintaining proper hygiene to reduce recurrence and enhance the quality of life. Further advancements in reconstructive techniques and microsurgical methods hold promise for improving patient outcomes in severe cases.
